# The FAIR Cookbook - the essential resource for and by FAIR doers

**DOI:** 10.1038/s41597-023-02166-3

**Published:** 2023-05-19

**Authors:** Philippe Rocca-Serra, Wei Gu, Vassilios Ioannidis, Tooba Abbassi-Daloii, Salvador Capella-Gutierrez, Ishwar Chandramouliswaran, Andrea Splendiani, Tony Burdett, Robert T. Giessmann, David Henderson, Dominique Batista, Ibrahim Emam, Yojana Gadiya, Lucas Giovanni, Egon Willighagen, Chris Evelo, Alasdair J. G. Gray, Philip Gribbon, Nick Juty, Danielle Welter, Karsten Quast, Paul Peeters, Tom Plasterer, Colin Wood, Eelke van der Horst, Dorothy Reilly, Herman van Vlijmen, Serena Scollen, Allyson Lister, Milo Thurston, Ramon Granell, the FAIR Cookbook Contributors, Gabriel Backianathan, Sebastian Baier, Anne Cambon Thomsen, Martin Cook, Melanie Courtot, Mike d’Arcy, Kurt Dauth, Eva Marin del Piico, Leyla Garcia, Ulrich Goldmann, Valentin Grouès, Daniel J. B. Clarke, Erwan Lefloch, Isuru Liyanage, Petros Papadopoulos, Cyril Pommier, Emiliano Reynares, Francesco Ronzano, Alejandra Delfin-Rossaro, Venkata Sagatopam, Ashni Sedani, Vitaly Sedlyarov, Liubov Shilova, Sukhi Singh, Jolanda Strubel, Kees van Bochove, Zachary Warnes, Peter Woollard, Fuqi Xu, Andrea Zaliani, Susanna-Assunta Sansone

**Affiliations:** 1 Oxford e-Research Centre, Department of Engineering Science, University of Oxford, 7 Keble Road, OX13QG Oxford, UK; 2 AstraZeneca, Data Office, Data Science & AI unit R&D, 136 Hills Rd, Cambridge, UK; 3 Luxembourg Centre for Systems Biomedicine, ELIXIR Luxembourg, University of Luxembourg, L-4367 Belval, Luxembourg; 4 Luxembourg National Data Service, 6 Avenue des Hauts-Fourneaux, Esch-sur-Alzette, Luxembourg, L-4362 Esch-sur-Alzette, Luxembourg; 5 Vital-IT Group, SIB Swiss Institute of Bioinformatics, 1015 Lausanne, Switzerland; 6 Department of Bioinformatics (BiGCaT), NUTRIM, FHML, Maastricht University, Maastricht, the Netherlands; 7 Barcelona Supercomputing Center (BSC), Plaça Eusebi Güell, 1-3, 08034 Barcelona, Spain; 8 Office of Data Science Strategy, National Institutes of Health, 9000 Rockville Pike, Bethesda, Maryland 20892 USA; 9 IQVIA, Kirchgartenstrasse 14, Basel, Switzerland; 10 European Molecular Biology Laboratory, European Bioinformatics Institute (EMBL-EBI), Hinxton, CB10 1SD UK; 11 Bayer AG, Business Development & Licensing & OI, Pharmaceuticals, 13342 Berlin, Germany; 12 Institute for Globally Distributed Open Research and Education (IGDORE), Berlin, Germany; 13 Data Science Institute, Imperial College London, William Penney Laboratory, South Kensington Campus, London, SW7 2AZ UK; 14 Fraunhofer Institute for Translational Medicine and Pharmacology and Fraunhofer Cluster of Excellence for Immune Mediated Diseases, Schnackenburgallee 114, 22525 Hamburg, and Theodor Stern Kai 7, 60590 Frankfurt, Germany; 15 Department of Computer Science, Heriot-Watt University, Edinburgh, EH14 4AS Scotland UK; 16 The University of Manchester, Department of Computer Science, The University of Manchester, Manchester, M13 9PL UK; 17 Boehringer Ingelheim Pharma GmbH & Co. KG, Birkendorfer Straße 65, 88397 Biberach an der Riss, Germany; 18 Janssen, Turnhoutseweg 30, B-2340 Beerse, Belgium; 19 AstraZeneca Pharmaceuticals, 36 Gatehouse Drive, Waltham, MA 02451 USA; 20 AstraZeneca, da Vinci Building, Melbourn Science Park, Cambridge Road, Royston, SG8 6HM UK; 21 The Hyve BV, Arthur van Schendelstraat 650, 3511 MJ Utrecht, The Netherlands; 22 Novartis Institutes for BioMedical Research, Novartis Pharma AG, Basel, Switzerland; 23 ELIXIR Hub, Wellcome Genome Campus, Hinxton, Cambridge CB10 1SD UK; 24 Novartis Institutes for BioMedical Research, Novartis Pharma AG, Cambridge, MA 02139 USA; 25 Bioinformatics and Information Technology Group, Leibniz Institute of Plant Genetics and Crop Plant Research, 06466 Seeland, Germany; 26 CNRS, Center for Epidemiology and Research in POPulation health, Université de Toulouse, Inserm, UPS, Joint Unit, 1295 Toulouse, France; 27 University of Southern California Information Sciences Institute, Marina del Rey, CA 90292 USA; 28 ZB MED, Information Centre for Life Sciences, Gleueler Straße 60, 50931 Cologne, Germany; 29 Center for Molecular Medicine, Lazarettgasse 14, AKH BT 25.3, 1090 Wien, Austria; 30 Department of Pharmacological Sciences, Mount Sinai Center for Bioinformatics, Icahn School of Medicine at Mount Sinai, New York, NY 10029 USA; 31 Université Paris-Saclay, INRAE, BioinfOmics, Plant bioinformatics facility, 78026 Versailles, France; 32 Hospital del Mar Institute of Medical Research, DCEXS, Pompeu Fabra University, 08003 Barcelona, Spain; 33 GSK - Stevenage, Gunnels Wood Rd, Stevenage, SG1 2NY UK

**Keywords:** Scientific community, Computational biology and bioinformatics

## Abstract

The notion that data should be Findable, Accessible, Interoperable and Reusable, according to the FAIR Principles, has become a global norm for good data stewardship and a prerequisite for reproducibility. Nowadays, FAIR guides data policy actions and professional practices in the public and private sectors. Despite such global endorsements, however, the FAIR Principles are aspirational, remaining elusive at best, and intimidating at worst. To address the lack of practical guidance, and help with capability gaps, we developed the FAIR Cookbook, an open, online resource of hands-on recipes for “FAIR doers” in the Life Sciences. Created by researchers and data managers professionals in academia, (bio)pharmaceutical companies and information service industries, the FAIR Cookbook covers the key steps in a FAIRification journey, the levels and indicators of FAIRness, the maturity model, the technologies, the tools and the standards available, as well as the skills required, and the challenges to achieve and improve data FAIRness. Part of the ELIXIR ecosystem, and recommended by funders, the FAIR Cookbook is open to contributions of new recipes.

## Introduction

The FAIR Principles^1^ have taken the world of scientific data management by storm, aligning stakeholders behind a common set of guidelines to ensure data (including datasets and software) is Findable, Accessible, Interoperable, and Reusable. The FAIR Principles describe characteristics for systems and services to make data a valuable asset, which could be rigorously evaluated, extensively reused, reproduced or repurposed, ensuring appropriate credit and legitimate use, to the benefit of both creators and users. The great merit of the FAIR movement has been to propel key, basic concepts of good data management worldwide, gaining acceptance in organizations from all sectors. Funding agencies are consolidating FAIR into their funding agreements, scholarly publishers have united behind FAIR as a way to promote and remain at the forefront of open research and FAIR is adopted and enshrined in guidances by libraries, scholarly societies and community alliances. In the Life Sciences, where FAIR was born, public and private organizations strive to implement the principles into practices to unlock data’s potential for future research. Major research infrastructure and governmental organizations, such as the pan-european ELIXIR (https://www.elixir-europe.org) and the USA NIH Office of Data Science Strategy (https://datascience.nih.gov), work to develop and fund resources, practices and policies that enable the collection, storage, analysis, use, and sharing of data according to FAIR practices. Major (bio)pharmaceutical companies value their data as an essential corporate asset, and consider FAIR as a key driver of their digital transformation; companies, providing services and solutions for research, also display FAIR in their portfolio^2,3^. Undoubtedly, FAIR has become essential in support of today’s data-driven scientific research, which has shifted towards automation, requiring the use of data at scale by machine, with minimal human intervention. In today’s world, large cohort studies and multimodal datasets are routinely produced and used, and the criticality of their data readiness, interoperability, and reusability has been acutely felt during the public health emergency of 2020–2021.

Despite the wide mobilization and adoption, there is a gap between stakeholders’ expectations and the guidance that is needed to execute them. In particular, there are two key challenges we must overcome to make FAIR a reality. Firstly, within any organization or project, often it is exceptionally difficult to plot a path to a FAIRer state. The FAIR Principles are aspirational guidelines, and not a formalized standard; also there is neither one specific technology to implement, nor one magic solution to follow. This means that FAIR is not the destination, but it is a continuum, a journey^4^, and each path to FAIRness can follow a different route. Also, there is a large body of generic FAIR guidance, which is high level and domain agnostic^5,6^, but lacking practical examples on “how to” for different data types and scenarios. Coupled with a digital skills shortage and talent gap to be filled, in all sectors, especially around research data stewardship, these factors make it difficult to confidently devise methods that will ensure data will be FAIR. Secondly, it is challenging to accurately evaluate the costs and benefits of FAIR data. To encourage budget holders to invest in FAIR, it is necessary to demonstrate the value of any proposed operational changes associated with the delivery of FAIR data and services; success stories, however, are usually anecdotal. Fostering change in culture around research practices^7,8^ depends on a clear statement of the types of improvement that can be achieved as well as on understanding when ‘FAIR enough’ has been reached, following a cost/benefit analysis^9–11^.

The FAIR Cookbook (https://faircookbook.elixir-europe.org) is our contribution towards addressing these two challenges. Launched in 2020, the FAIR Cookbook was created collaboratively and pre-competitively by academics, major (bio)pharmaceutical companies, and information and service companies partners in FAIRplus (https://fairplus-project.eu). This is an international project funded by Innovative Medicine Initiative (IMI, https://www.imi.europa.eu now Innovative Health Initiative, IHI), the largest private-public European partnership program funding health research and innovation. In this paper, we present the FAIR Cookbook, focussing on its creation and content, its value, use and adoptions, as well as the participatory process, and collaborative plans for sustainability, which have already moved beyond the initial project.

## Results

### Use it, adopt it, join it: A practical guide in the FAIRification journey

Created by researchers and data managers professionals from the public and private sectors, the FAIR Cookbook is an online resource that delivers content (“recipes”) to help users make and keep data FAIR, with a specific focus in the field of Life Sciences. The technical infrastructure, detailed in the Method section, is built on open source community practices. The recipes cover the operation steps of FAIR data operations and services. The FAIR Cookbook helps users achieve and improve data FAIRness, by learning how to FAIRify datasets, use levels and indicators of FAIRness, discover the appropriate technologies, tools and standards, as well as understand the skills required, and the challenges. The FAIR Cookbook is a live resource because recipes are added and improved, iteratively, and peer-reviewed in an open manner.

The two key objectives of the FAIR Cookbook are to fill the current gaps between high-level FAIR Principles and their actual implementation, and contribute to the necessary culture change to deliver FAIR in public and private organizations. The FAIR Cookbook is for all stakeholders in the data life cycle in Life Science, who aim to put FAIR into practice, as summarized in Text box 1.

Box 1 The FAIR Cookbook is primarily a resource for “FAIR doers”, but it also provides something to each stakeholder, from professionals working in research data management, to those creating guidelines, policies and educational material.

- To researchers and data stewards:
  - It provides some introductory material, and hands-on, technical step-by-step examples for an effective FAIRification journey.
- To policy makers and trainers:
  - It delivers practical examples to recommend in policies, and use in educational material to incentivize and guide FAIR in practice.
- To data managers and technical professionals:
  - It offers a venue to document and share existing and new approaches or services to support FAIRification, via recipes that show exemplar FAIRified datasets, FAIRification tools, workflows, and other resources.
  - It also promotes a participatory culture that enables sharing of expertise by getting exposure and credit, and contributes towards building organizations’ FAIR capacity.

### Live recipes: A combination of guidance, hands-on, and examples

As of February 2023, there are over 82 production-grade recipes in the FAIR Cookbook. The FAIR Cookbook recipes are a combination of guidance, technical, and hands-on instructions, and are organized in two main groups. The first is designed around the FAIR Principles, covering technical and operational processes and applicable to any data types. The second covers three specific topics: (i) software infrastructure to build FAIR processes, such as ontology browsers or data catalogs; (ii) the process of assessing FAIRness using well known tools^12,13^; and (iii) examples of FAIRified datasets in the molecular, pre-clinical and clinical areas. The latter were developed working with data producers in IMI/IHI projects and (bio)pharmaceutical companies, and provide users with exemplar cost-effective FAIRification pathways in specific contexts.

The concept of a recipe was first prototyped and published as an article^14^, and subsequently converted into a proper record in the FAIR Cookbook (http://w3id.org/faircookbook/FCB037). Persistent unique identifiers are used to cite recipes and credit their authors, and the content is released under an open license. Graphical representations are used, where needed, for illustrative purposes. Links or cross-links to complementary online resources provide additional reading material, as relevant. About a fifth of the current recipes have associated executable code, which can be run on cloud based infrastructures. All technical features are detailed in the Method section.

In each recipe, a summary card displays the reading time, level of difficulty, and audience types, as detailed in the Method section. For instance, recipes for researchers include those on how to choose licenses for datasets, package data for transfer, and provide sufficient descriptive metadata. Recipes for data stewards and managers include those providing exemplar FAIRification datasets, and how to deal with sensitive, patient-centric data. Recipes for technical professionals include those focusing on a number of FAIR-enabling tools and services, and on how to manage terminologies and data curation services. The recipe’s summary cards also display maturity levels and indicators, described in the Method section, which helps users target a maturity level that aligns with an anticipated FAIRification goal. This feature enables the users to assemble recipes, building a coherent path, which follows the data management expectations in terms of FAIRness.

### Which recipes: Where and how to start

The FAIR Cookbook is designed to be useful to a variety of users and scenarios. From the home page, the users can either seek information on a specific FAIR topic directly, or they can browse and select relevant recipes via the search function (https://faircookbook.elixir-europe.org/content/search-wizard.html). In addition, the FAIR Cookbook also includes a forewords section with recipes that provide more general guidance, for example, on the ethical values of FAIR (https://w3id.org/faircookbook/FCB072) on how to prioritize which project, process or data to FAIRify (https://w3id.org/faircookbook/FCB055), or considerations on what metadata is and how many types there are (https://w3id.org/faircookbook/FCB068). Once users have identified their goals, they can focus on relevant recipes in the sections on the FAIR components, as illustrated in Text box 2, or in the sections on the software infrastructure, the assessment tools, and the exemplar FAIRified datasets.

The FAIR Cookbook also provides, in a dedicated section, a number of recipes with applied examples of different goals using different data types, (https://faircookbook.elixir-europe.org/content/recipes/applied-examples.html). These recipes describe real life FAIRification processes in a number of IMI/IHI research projects. If users need generic guidance on the FAIRification processes, we describe in a sister paper^15^, a framework that helps them to: (1) set realistic and practical goals; (2) examine data, capability and resource requirements; (3) assess, design, implement and reiterate; (4) review against the initial goals. This FAIRification process is also outlined in a recipe (https://w3id.org/faircookbook/FCB079).

Box 2 Examples of goals and recipes, in the relevant sections, which assist towards meeting the needs.

- **Goal**: improving visibility of content
  - Recipes: on identifiers (https://w3id.org/faircookbook/FCB006), chemical structures (https://w3id.org/faircookbook/FCB007) and search engine optimization (https://w3id.org/faircookbook/FCB010), under Findability.
- **Goal**: semantic integration of datasets from multiple sources
  - Recipes: selecting terminologies and ontologies (https://w3id.org/faircookbook/FCB020) and an ontology service (https://w3id.org/faircookbook/FCB004), under Interoperability.
- **Goal**: security compliance and with regulators
  - Recipes: on secure data transfer protocol (https://w3id.org/faircookbook/FCB014), under Accessibility;
  - declaring data’s permitted use (https://w3id.org/faircookbook/FCB035), under Reusability.

### Contributions and synergies: Cultivating the collective knowledge

As of February 2023 the contributors to the FAIR Cookbook number almost 100 researchers and data managers professionals from more than 40 academic and industrial organizations, across the wider ELIXIR network, and beyond. This ensures diversity and coverage of topics and skills. All authors are FAIR doers, for example, being involved in data management projects, or being in charge of technical or curatorial resources, or being the provider of such services. Our editorial and operational processes include a number of routes to contribute content asynchronously, as detailed in the Method section, and book dash events. The latter are short (one or half) day online events where people come together to work on recipes; the schedule generally includes onboarding, goal setting, discussion and writing sessions. We built a thriving contributing community by leveraging on three motivational points: (i) the need to stay engaged, and updated with the latest developments; (ii) the value of being visible in the FAIR space, and recognized as an expert; and (iii) the importance of expanding the network of collaborators, clients, or users. In many cases, the creation of recipes offered opportunities to unlock the collective knowledge towards synergistic approaches. For example, different groups and projects come together to harmonize the practices around the FAIRification of observational studies and databases, which were captured as a recipe (https://w3id.org/faircookbook/FCB054).

The content creation process was mainly informed by the needs of IMI/IHI data-producing projects and the participating (bio)pharmaceutical companies. For example, colleagues at Novartis AG contributed insights to ensure that Contract Research Organizations (CROs) deliver data that is as FAIR as possible to the paying contractee; the result was a new dedicated recipe (http://w3id.org/faircookbook/FCB056). Another specific contribution, however, came from the members of the FAIRplus Fellowship Programme (https://fairplus-project.eu/get-involved/fellowship), which has now concluded, in the form of improvements to the recipes. This training program for 20 individuals (from academia and industry) was set to empower them to become FAIR ambassadors in their respective organizations. Using their own datasets, and with their own goals and challenges, the FAIRplus fellows reviewed the FAIR Cookbook to identify gaps in coverage, or areas where content needed improvements or extensions. For instance, a deeper coverage of the notion of “provenance information” was requested, along with clarifications about the different types of metadata: structural metadata, administrative metadata, provenance metadata or quality metadata. The provenance and metadata aspects were improved, and covered by two specific recipes (https://w3id.org/faircookbook/FCB036 and https://w3id.org/faircookbook/FCB068), respectively. Current topics of interest and focus are recipes around knowledge graphs, applications of semantic web standards, and more examples of FAIRified datasets.

### Value and use: Educational, every-day tasks and strategic purposes

The latest statistics show that in the past 12 months (January 2022-January 2023), 13,889 total users accessed the FAIR Cookbook, totaling 50,418 page views (source Google Analytics). More importantly, we validated the utility and value of the FAIR Cookbook based on three uses: (i) as an educational resource on FAIR in a training context; (ii) as practical guidance on how to improve day-to-day tasks for FAIRer data; and (iii) as a contributor towards changing the culture in research data management. In the sections below, we summarize our findings.

The FAIR Cookbook was used as educational material in the context of the FAIRplus Fellowship Programme^16^; by design fellows were both consumers of existing recipes, and producers of novel ones. The FAIR Cookbook helped the fellows gain a deeper understanding in terms of FAIR know-how, and apply the gained expertise to their own data, and in real scenarios. The fellows largely reported that they achieved the expected results, and their experience was a direct observable confirmation of the validity of the recipes’ content towards the intended (learning) objectives. There is no denying that some recipes require a greater amount of technical background knowledge, and a steeper learning curve, a fact that is illustrated on recipe cards, at the top of each recipe, as detailed in the Method section. Due to the wide variety of backgrounds among the FAIRplus fellows, some technical content was indeed challenging for some; however, working in pairs helped to optimize information sharing and the learning process. Following this successful experience, we are in the process of embedding the FAIR Cookbook into other relevant programs set to ‘training the trainers’. An example is the FAIR Data Steward Ambassadorship program, part of the ELIXIR-UK Node (https://elixiruknode.org/projects/elixir-uk-dash), which is building a community of data stewards with practical experience in contemporary life science data management in the UK.

Working with the partners in FAIRplus, we assessed the utility of the FAIR Cookbook in (bio)pharmaceutical companies, where the shift of the data management culture to a FAIR practice represents one of the biggest internal data science bets for these enterprises. The assessment, as well as any subsequent operations, happened behind their firewalls. Therefore, the three outcomes we report here were expressed in terms of satisfaction of the value of the recipes, against specific tasks, or challenges addressed. In the first example, colleagues at Janssen reported a positive contribution of the FAIR Cookbook towards their discussion on return on investment to operationalize FAIR.

Janssen’s specific interest was in project asset continuity, and any associated metric that allows to gauge the sustainability and impact of their projects. In particular, implementing procedures that improve, in measurable ways, data asset mobilization and reuse, justifying the necessary efforts and investments. Janssen’s goal was to evaluate the design of a proposed internal data lake system for hosting a large data set. The FAIR Cookbook served as guidance to assess the FAIRness of the data both in its initial state, i.e., a collection of numerous files, and in the more structured state that would be achieved after the implementation of the data lake. In the initial file-based state, gaps were identified such as the lack of appropriate structure in the file format used, the absence of rich metadata relevant to its interpretability, an over-reliance on human-readable identifiers, and the inadequacy of the data access mechanism. All these aspects limited the re-use of the data. The assessment of the projected future state concluded that there would be a substantial improvement by enforcing a better standardization, by having stronger links with other corporate databases, and by having a unified data access mechanism. These considerations helped Janssen to build a consensus on the optimal solution among the developers of the system, and to justify the investment in the required improvements. The FAIR Cookbook may not yet contain recipes for all elements involved in a large pharmaceutical drug discovery operation, but it still offered very useful guiding principles for how such gaps could best be addressed. Janssen learned how to, in a complex data space, get a strategic overview of what are the most crucial challenges from a cost-benefit perspective, referring to the maturity levels and indicators displayed in each recipe. Janssen benefited from the lessons on how to run an agile and iterative FAIRification process internally, and intends to leverage on the materials and insights and integrate the lessons learned.

In the second example, collaborators from Boehringer Ingelheim reported a positive impact of the FAIR Cookbook. They demonstrated internally the power of using open ontologies for performing data integration tasks (https://w3id.org/faircookbook/FCB022), and of building application ontologies (https://w3id.org/faircookbook/FCB023) using an open-source tool and a set of semantic resources, which are open and interoperable by design, from the Open Biomedical Ontologies Foundry^17^. In the third example, colleagues at AstraZeneca requested guidance and best practice to make metadata (data usage rights) FAIR, for an implementation based on DCAT (https://www.w3.org/TR/vocab-dcat-3); this was achieved via a representation based on the Resource Description Framework standard. The result was the joint creation of a dedicated recipe on digital rights (https://w3id.org/faircookbook/FCB035), which now influences clinical data usage policy by shaping clinical data access rights in our enterprise data registry. Lastly, working with a number of IMI/IHI data-producing research projects, we applied relevant recipes to FAIRify their datasets. We documented the process in recipes that now serve as exemplars (https://faircookbook.elixir-europe.org/content/recipes/applied-examples.html) to other users with similar goals and data types.

### Adoptions and collaborations: A flagship resource in life science

We were also successful in reaching a considerable level of international support for and endorsement of the FAIR Cookbook by major research infrastructures, pre-competitive initiatives and governmental organizations, in a very short time. This reflects the quality, maturity and unique value of this resource. In particular, at European Commission (EC) level, the FAIR Cookbook is recommended by the IMI/IHI Project Guidelines for “Open Access to Publication and Research Data^18^, and by the EC report on FAIR data for a coordinated COVID-19 response^19^. A recent and major milestone, which reflects its standing in the community, is its addition to the Horizon Europe Work Programme 2023–2024 for Health^20^.

Featured as one of the achievements in ELIXIR Annual Report in 2021, in the same year, the FAIR Cookbook became an ELIXIR service provided by the UK and Luxembourg Nodes, followed by the Switzerland Node in 2022, and the ELIXIR Spain Node in 2023. Becoming a Node-provided service is the result of a formal review process that looks at the quality, maturity, use and impact of the proposed resource, and this is also the ELIXIR’s formal instrument to ensure the collaborative maintenance and sustainability of highly valuable shared resources. Nowadays, the FAIR Cookbook is among the ELIXIR flagship resources (https://elixir-europe.org/what-we-offer/guidelines), and it is embedded in the new ELIXIR Scientific Programme 2024–2028.

Working as part of a growing ecosystem of FAIR resources, we also established collaborations with other initiatives and integrated its content in diverse online guidelines. For example, we started to progressively create bi-directional links between sections of the the step-by-step instructions in the recipes to other reference material, such the RDMkit (https://rdmkit.elixir-europe.org/index.html), another ELIXIR resource for the data management practices, and the Pistoia Alliance’s FAIR Toolkit (https://fairtoolkit.pistoiaalliance.org), an industry oriented, pre-competitive entry-level guidance to FAIR. Other global resources are referenced via mono-directional links, for example, recipes links to records in the multidisciplinary FAIRsharing^21^ (https://fairsharing.org) for more details on standards (reporting requirements, terminology artifacts, models, formats, identifier schemas), repositories (databases and knowledge bases) and data policies. Work is in progress also to tag recipes with a terminology that describes the competencies, skills and knowledge associated with making and keeping data FAIR (https://www.eoscsecretariat.eu/cocreating-eosc/terms4FAIRskillsPOC).

The FAIR Cookbook was designed from the start for long term sustainability by the ELIXIR network, with contributions via private-public-partnerships, and key collaborators from other major Life Science organizations. This strategy for sustainability is yielding. One notable example is the collaboration with the USA NIH Office of Data Science Strategy, where the Program Director, who oversees the establishment of a FAIR data ecosystem, has become a member of the FAIR Cookbook’s Editorial Board (faircookbook-ed@elixir-europe.org). This can potentially enable global knowledge exchange to enhance the impact of the FAIR Cookbook recipes, allow for the exploration of joint activities, such as book dash events, and the development of best practice recipes from USA experts to grow the network of FAIR doers.

## Discussion

Created and managed by professionals who work every day with data in academia, (bio)pharmaceutical companies and information service industries, the FAIR Cookbook is a live, open and collaborative resource, which documents the fundamental processes and capabilities for provisioning FAIR data and services. The citable recipes are developed collaboratively by credited authors, and anchored to real examples and use cases by data-producing projects and organizations. We illustrated how the FAIR Cookbook is uniquely positioned not only to serve as practical guidance to improve every-day tasks, but also to contribute to a curriculum on FAIR data, and inform discussions around the necessary changes to deliver FAIR within organizations. The FAIR Cookbook is a resource for all stakeholders involved in the data life cycle. No single group can cover all topics, because “it takes a village”^22^ to deliver FAIR in practice. That is why we have also devised ways to ensure anyone can highlight missing topics, and other experts can contribute, as detailed in the Method section, to the expansion of the content in terms of breadth and depth, as well as filling gaps.

From the onset, we looked at a multi-layered approach to ensure the sustainability of the FAIR Cookbook. As for any resource developed by funded projects, the risk is to be short-lived; long-term sustainability is the common challenge, regardless of how successful a resource becomes. Our approach to sustainability for FAIR Cookbook follows four fronts: (i) infrastructure, (ii) content, (iii) embedding and (iv) endorsements. The technical infrastructure is lightweight, off the shelf with a light hosting footprint, and the code is open; the website has an ELIXIR sub-domain name. The infrastructure’s key strengths are its low maintenance and FAIRness as an online resource, as detailed in the Method section. Key challenges are the implementations of new functionalities, but these could be created as part of newly funded projects, which the FAIR Cookbook is included in.

As per content sustainability (the most critical aspect), this is currently a distributed responsibility across the contributors and the Editorial Board, whose current membership is with ELIXIR Nodes and NIH representatives. The FAIR Cookbook has quickly become a catalyst for FAIR doers, successfully unlocking the collective knowledge around each FAIR topic. Key strengths helping content sustainability are its specialized and detailed information, which is also crosslinked to complementarity resources, to reduce duplication and increase traffic; and its citable recipes, credited to authors, motivating contributions. Key challenges are that, although virtually anyone can contribute content, the tasks of authoring and reviewing recipes, as well as keeping them up-to-date, requires commitment, hands-on expertise, and time. On the embedding and endorsement fronts, the FAIR Cookbook showed a strong start: it is already recommended by funding agencies, and it is rooted in major infrastructure organizations. Its role in ELIXIR will enable the FAIR Cookbook to grow as part of the ELIXIR framework of FAIR services. Its collaboration with the Pistoia Alliance (https://www.pistoiaalliance.org) will facilitate the continued collaboration with (bio)pharmaceutical companies and companies that provide services and solutions for research. Furthermore, the participation of the USA NIH Office of Data Science Strategy will be pivotal to expand the operations by involving and serving more academics and private partners for the community’s good. The key strengths of the embedding and endorsement fronts are that the FAIR Cookbook is a recognized unique resource of high-quality value, anchored to users’ needs; and it has already gained the trust of and the support from key international organizations. The key challenges are to establish a governance and coordination structure that scales up and meets the expectations of a growing community of collaborators.

Building the FAIR Cookbook, we made early design decisions to focus on expanding the content, as well as filling gaps and identifying additional resources that should be cross-referenced. The FAIR Cookbook has also succeeded in activating two main discussions around how to enable further expansion and management of the content in specialized domains, e.g. for plant science, and by the private sector, which can share what is pre-competitive but needs to protect what is proprietary. To meet the interest of the specialized communities, we will explore the creation of Domain Boards, which can be responsible for the coordination of the contributions within their domain community. To serve the needs of the private sector and benefit from their contributions, we will explore the concept of a network of FAIR Cookbook instances, with a public instance and internal versions for organization-specific recipes, for example regarding security and data access topics. Both routes (the Domain Boards and the network of FAIR Cookbook instances) will also contribute towards the sustainability challenges and contribute to the previously mentioned sustainability strategy encompassing infrastructure, content, embedding and endorsements.

As the FAIR Cookbook recipes are progressively embedded in training material and courses, we will receive more feedback from users and insights on their experience, which we plan to collect in a more systematic way. To improve the user journey, our next steps will include adding more guidance on the maturity level and indicators, to illustrate how each recipe advances the user along the FAIR continuum, as well as improving the searches by goal and by user type. The latter, in particular, will be coordinated in ELIXIR with the objective to have ‘a set of common paths’ into a number of complementary FAIR resources, to fulfill a number of scenarios and stakeholders needs.

We firmly believe that the success of the FAIR Cookbook is due to the timely delivery of specialized content, which fills the glaring gap between high-level FAIR Principles and their actual implementation in the Life Sciences. Our long term goal is to make FAIR less intimidating to real world end users. Our recipe for success is based on crediting expertise and contributions, promoting convergence and collaborations, and acknowledging the diversity of approaches towards the desired level of FAIRness. In a relatively short time, the FAIR Cookbook has attracted international contributors and users outside the project that directly funded its creation. What about you? Use it, contribute to it, and share it!

## Method

The FAIR Cookbook relies on well-established off-the-shelf solutions, frameworks and open source community practices to minimize development and maintenance costs. In the following sections, we detail the approach followed ranging from the infrastructure selection and content creation to publication methods.

### Technical infrastructure

The Cookbook is built using Jupyter Book^23^, following the example of The Alan Turing Institute’s “The Turing Way Book of Data Science”^24^, an open source community-driven generic guide to reproducible, ethical, inclusive and collaborative data science. The technology stack includes: GitHub (https://github.com) for version control, hosting, continuous integration, and automation of the build (via custom GitHub Actions); Jupyter Book engine (https://jupyterbook.org); written materials in markdown; HackMD markdown editor (http://hackmd.io), integrated with GitHub; Jupyter Notebooks for executable code^25^; binder for the web execution of Jupyter Notebook distributed with a recipe.

### Framework and syntax

For content management, we used Jupyter Book which allows content to be written in markdown. To ease engagement with contributors, the HackMD extension for the Chrome web browser was used to write, edit, and collaboratively review the content generated by the contributors. In addition, two more contribution routes were added: a less technically involved path, relying on shared Google documents, and one for technically advanced users who can use integrated development environments to write markdowns or notebooks and to commit to the GitHub repository branches, as they would with code. For both contribution mechanisms, recipe templates were devised. Lastly, computational notebooks in the form of native jupyter notebooks are seamlessly integrated in the Jupyter Book infrastructure and can be executed on either Binder (https://mybinder.org) or Google Colaboratory (https://colab.research.google.com)^26^ cloud infrastructure at the click of a button.

### Visual identity and standard layout

**Fig. 1:**
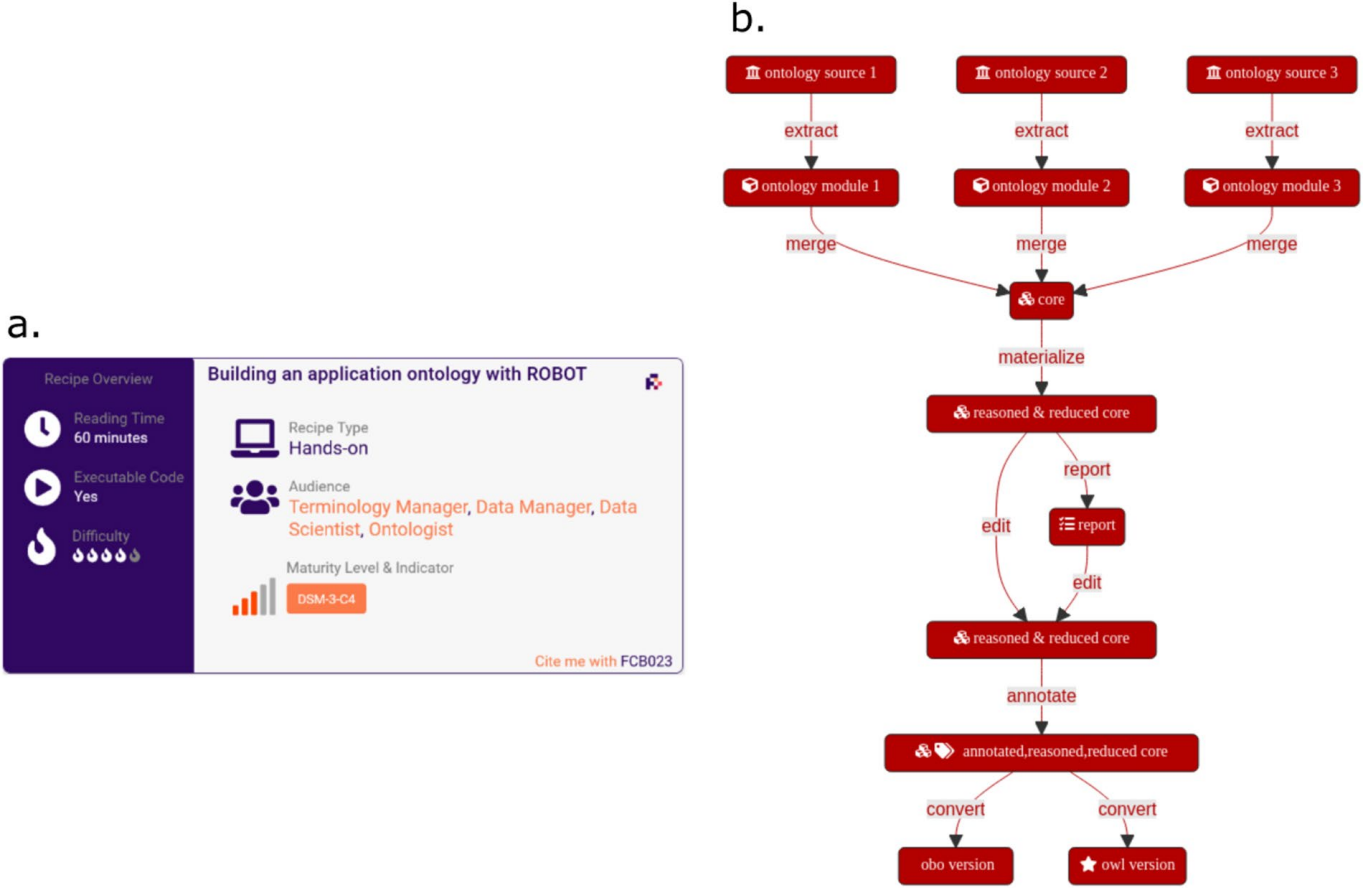
(**a**) A summary card of a FAIR Cookbook recipe (https://w3id.org/faircookbook/FCB023) showing key information. A machine-readable summary metadata is available as a JSON-LD markup embedded in the HTML page. (**b**) A mermaid generated flowchart diagram providing an overview of the key steps of the procedure described in the recipe, which in this case describes how to build an application ontology.

Standard web techniques were used to develop a distinctive visual identity for the FAIR Cookbook and sections of the recipes, such as the ‘summary cards’; icons were provided by the Font Awesome 5.0 free and open version (https://fontawesome.com). The Mermaid JavaScript library (https://github.com/mermaid-js/mermaid) was used for flowcharts, charts and class diagrams. The Mermaid live editor (https://mermaid-js.github.io/mermaid-live-editor/#) was used to collaboratively create diagrams during knowledge elicitation steps. Images were standardized and files saved as MMD files before being rendered to PNG format. An example is shown in Fig. 1. Recipes were structured around a standardized layout, to assist the reader, but also to facilitate the review and quality control process.

### Citability of recipes and credit to authors

**Fig. 2:**
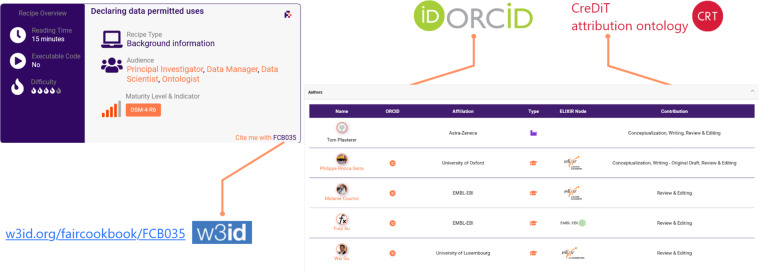
Citability of recipes and identification of and credit for authors; an example is provided.

Following the evaluation of various persistent identifier services, (e.g. https://fairsharing.org/standards/identifier_schema), the w3id approach (https://w3id.org/) was selected based on ease of use and cost effectiveness (https://w3id.org/faircookbook/FCB077). Furthermore, to incentivize and credit contributors we enabled the use of ORCID^27^, and of the CreDiT ontology^28^ to specify author’s roles. An example is shown in Fig. 2. Furthermore, the FAIR Cookbook resource itself was registered in identifiers.org^29^, with its own dedicated namespace (https://registry.identifiers.org/registry/fcb) to enable the content to be replicated in multiple resources under a single unifying URI, facilitating downstream integration.

### FAIR maturity indicators

**Fig. 3:**
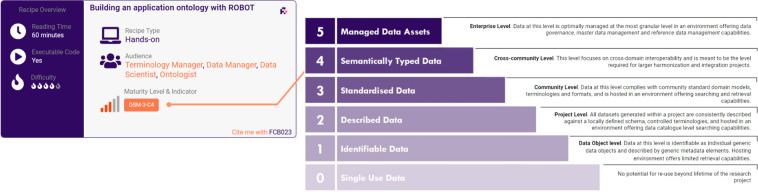
Recipe card showing the DataSet Maturity indicators and corresponding maturity level. In this instance, the indicators DSM-3-C4 cover aspects of data and content (C) standardisation, in contrast to hosting (H) and representation (R).

The recipes were integrated with the FAIR DataSet Maturity (FAIR-DSM) model (https://fairplus.github.io/Data-Maturity), another output of the FAIRplus project. The FAIR-DSM indicators specify which maturity level(s) and aspect they measure (content, representation or hosting) may be achieved following the full extent of the recipe; one or more recipes may be needed to fulfill a maturity level. Figure 3 shows how each recipe has been anchored to the FAIR-DSM model and how this is represented. Depending on the recipe type, one or more indicators may apply.

The FAIR-DSM model was developed in parallel to the FAIR Cookbook and the integration was realized via a dedicated editorial review process, which required the authors of the recipe to ensure that the most relevant indicators were used. This integration allows exploration of the FAIR Cookbook also according to the desired improvements.

### Search engine optimisation

The Jupyter Book infrastructure provides minimal support for allowing content to be indexed by search engines, therefore, we augmented it relying on the sphinx-sitemap extension to generate the sitemap.xml file, which is required by search engines to crawl a website. Since findability is essential to all online resources, this method is also illustrated in a specific “Search engine optimization” recipe (https://w3id.org/faircookbook/FCB010). In addition, each recipe’s content page was marked up via custom Python extension code, which harvests key metadata to generate a machine-readable description using the Bioschemas Training Material profile (https://bioschemas.org/profiles/TrainingMaterial/1.0-RELEASE)^30^ and the schema.org *HowTo* *Type* (https://schema.org/HowTo). This delivers recipes metadata in JavaScript Object Notation for Linked Data format (JSON-LD, https://json-ld.org) that is added to the HTML head section of each recipe’s page.

### Search wizard

**Fig. 4:**
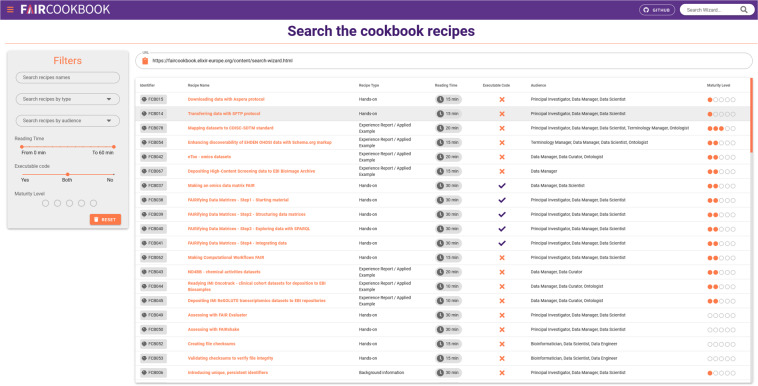
A searchable view of the FAIR Cookbook content, via the search wizard.

The searchability of the recipes was improved with the inclusion of a search wizard (https://faircookbook.elixir-europe.org/content/search-wizard.html), a dedicated interface and a set of filters, as illustrated in Fig. 4.

Jupyter Book is a Sphinx distribution (https://www.sphinx-doc.org) and comes bundled with a simple search engine with limited findability and discoverability. This neither offers granular filtering and sorting systems, nor does it display metadata about the recipes. Therefore, we developed a custom tool to search and filter recipes by name, type, audience, reading time, maturity level, and whether they contain executable code; it also delivers a stable URL for each search query to allow users to share the results of a search. The search wizard relies on the metadata in the recipe’s summary cards, but when users cannot find a recipe matching their search criteria, they are directed to use a slightly enhanced version of the default search engine, which searches over all text. The search wizard will be improved based on users’ feedback, but it has already provided better navigability and findability of the recipes.

### The FAIRness of the FAIR Cookbook

The FAIR CookBook is also a FAIR resource itself, and this were made possible by the use of the following technologies and standards:

- Findability: (i) each recipe is identified via a unique persistent identifier provided by w3id.org; (ii) metadata is implemented via standards, such as schema.org and Bioschemas, and the identity of authors is based on ORCID identifiers; (iii) indexing is guaranteed by the search engines optimization via sitemap.xml and JSON-LD.
- Accessibility: Via a publicly-accessible website (HTTPS protocol).
- Interoperability: (i) JSON-LD markup; (ii) cross-links to records in other registries, as relevant; (iii) attribution roles to authors via the CreDiT ontology.
- Reusability: Creative Commons Attribution 4.0 International (CC BY 4.0) license.

### Editorial and review processes

The development of the FAIR Cookbook is supervised by an Editorial Board, which steers content creation, identifying areas where contributions are needed, tracking progress, and assisting with the review and content production processes. Members of the Editorial Board also worked with technical staff to ensure failsafe procedures by limiting the effects of technology dependency, testing the migration of different frameworks and hosting platforms. During the initial phase of the work, and to define the content skeleton, we created a Section Board. This was recently rescinded to plan for the creation of Domain Boards, which could coordinate and drive the expert creation of new content specific to Life Science areas.

The review process operates very much like that of a scholarly scientific publication. Both authors’ and reviewers’ contributions are credited in the recipe, which receives a unique identifier and is released to production. Reviewers are assigned and given a set of criteria to appraise the recipe (including coverage, syntax compliance, language, code presence and reproducibility/execution). The mechanisms to provide feedback to authors depend on the route used to submit the recipe, and generally include: direct email communication, github revision history and issue tracking, dash events, or one to one interaction in a dedicated teleconference. A systematic feedback mechanism is currently being developed, and we plan to make these functions available in a future release of the FAIR Cookbook.

### Content creation and operations in the building phase

To identify the topics for the recipes, a combined *top-down* and *bottom-up approach* was used in the building phase during the FAIRplus project. The former resulted in the creation of a prospective table of content, which identified themes that appeared key to the FAIR Cookbook developers. In the latter, the content was triaged for prioritization based on the needs collected from the IMI/IHI data-producing projects and the (bio)pharmaceutical companies. The triage worked by mapping the use cases along the FAIRification path^15^, and then the procedures were broken down into the smallest possible units to create recipes, which were revised and reviewed. Fortnightly one-hour calls were held to track progress and raise issues, while monthly content creation events, known as ‘book dashes’, were aligned with GitHub milestones to ensure a steady pace of the development. Major content reviews were performed during quarterly events as part of a group activity done by working under a three month-long “scrum” schedule, and using weekly calls to assess progress and exchange feedback. Slack communication, emails and the GitHub infrastructure were exploited to the full to enable and track the discussion. A Code of Conduct is also in place to ensure respectful and healthy interactions. Last, but not least, we created guidelines (https://faircookbook.elixir-europe.org/content/recipes/help.html) on what a recipe should or should not be, as summarized in Text box 3.

As we transition out of the FAIRplus project, and enter a maintenance and growth phase, we are working to revise the operations, which will take in account of the Domain Boards, the participation of the ELIXIR Nodes, as well as the collaboration with the USA NIH Office of Data Science Strategy, the Pistoia Alliance and any other interested organizations.

Box 3 Overview principles of what recipes should and should not be.

**Table:** 

A recipe should be		A recipe should not be	
Specific	Target a specific task or action or tool and service that brings FAIRness one or more levels up; or provide an example of data FAIRification.	Too broad and high level	Should not be a repeat of the full user manual.
			Should not be a features list of a tool.
Complete	Should be an end-to-end recipe that users can follow and finish a task.	Incomplete	Should not be just a teaser that only shows a few steps at the beginning.
			Should not be an advertisement.
Open	The tools and services used should be open, or, if proprietary, a “free” or “community” version should be available.	Closed	Users can only test it after purchasing a paid software.

### Release process and containerization

Continuous integration and release workflows were set up to automate the tasks and further increase end-user-friendliness. Furthermore, the integration of Zenodo with the GitHub infrastructure made it possible to automatically generate digital object identifiers with each new version release of the FAIR Cookbook. Using the GitHub functionality to cite a code repository independently from each new release, we added metadata formatted according to citation file format (https://github.com/citation-file-format/citation-file-format), which is in compliance with software citation principles^31^. Both aspects complement the stable w3id identifier minting for individual recipes. Finally, cited “collections” of related content can be populated under Zenodo “community” collections for highlighting specific project outputs in accordance with targeted audience findability and reusability. A Docker-based (https://docker.com) version of the FAIR Cookbook is available from the repository, enabling straightforward deployment. The containerization simplifies the interaction and local testing by authors prior to contributing content back to the code repository. It also eases on-premises deployment following content customization as is often the case with industrial partners.

## Data Availability

The FAIR Cookbook is published at https://faircookbook.elixir-europe.org. The content is available in a dedicated public repository on GitHub: https://github.com/FAIRplus/the-fair-cookbook, under the CC-BY-4.0 license.

## Code availability

The code is open source and available in a dedicated public repository on GitHub: https://github.com/FAIRplus/the-fair-cookbook.
